# Genetically engineered eucalyptus expressing pesticidal proteins from *Bacillus thuringiensis* for insect resistance: a risk assessment evaluation perspective

**DOI:** 10.3389/fbioe.2024.1322985

**Published:** 2024-03-18

**Authors:** Dror Avisar, Alexandre Manoeli, Anselmo Azevedo dos Santos, Antonio Carlos Da Mota Porto, Carolina Da Silva Rocha, Edival Zauza, Esteban R. Gonzalez, Everton Soliman, José Mateus Wisniewski Gonsalves, Lorena Bombonato, Maria P. Galan, Maurício M. Domingues, Murici Carlos Candelaria, Reginaldo Mafia, Rodrigo Neves Graça, Shelly Azulay, Sivan Livne, Tatiane Buono Dias, Thaís Regina Drezza, William Jose Silva, Ana Cristina Pinheiro

**Affiliations:** ^1^ FuturaGene Israel Ltd. (R&D), Rehovot, Israel; ^2^ Suzano S.A. (FuturaGene—Biotech Division), Itapetininga, Brazil; ^3^ Suzano S.A, SãoPaulo, Brazil; ^4^ Independent Researcher, Itapetininga, Brazil; ^5^ W. J. Silva Consultoria Agrícola S/C LTDA, Jardinópolis, Brazil

**Keywords:** eucalyptus, genetically modified, *Bacillus thuringiensis*, Cry pesticidal proteins, insect resistance, *Thyrinteina arnobia*, biosafety

## Abstract

Eucalyptus covers approximately 7.5 million hectares in Brazil and serves as the primary woody species cultivated for commercial purposes. However, native insects and invasive pests pose a significant threat to eucalyptus trees, resulting in substantial economic losses and reduced forest productivity. One of the primary lepidopteran pests affecting eucalyptus is *Thyrinteina arnobia* (Stoll, 1782) (Lepidoptera: Geometridae), commonly referred to as the brown looper caterpillar. To address this issue, FuturaGene, the biotech division of Suzano S.A., has developed an insect-resistant (IR) eucalyptus variety, which expresses Cry pesticidal proteins (Cry1Ab, Cry1Bb, and Cry2Aa), derived from *Bacillus thuringiensis* (Bt). Following extensive safety assessments, including field trials across various biomes in Brazil, the Brazilian National Technical Commission of Biosafety (CTNBio) recently approved the commercialization of IR eucalyptus. The biosafety assessments involved the analysis of molecular genomics, digestibility, thermostability, non-target organism exposure, degradability in the field, and effects on soil microbial communities and arthropod communities. In addition, *in silico* studies were conducted to evaluate allergenicity and toxicity. Results from both laboratory and field studies indicated that Bt eucalyptus is as safe as the conventional eucalyptus clone for humans, animals, and the environment, ensuring the secure use of this insect-resistant trait in wood production.

## Introduction

Genetically modified (GM) crops expressing *Bacillus thuringiensis* (Bt) proteins have led to major advancements in crop protection and productivity (Brookes and Barfoot, 2020; ISAAA, 2020). Since their commercialization in 1995, Bt crops have reduced chemical insecticide usage by over 50% while maintaining high yields and reducing greenhouse gas emissions (Brookes and Barfoot, 2018). Integrating Bt genes into crops represents a deviation from the reliance on chemical insecticides, which often cause environmental and health concerns and allow initial pest damage since application occurred after monitoring (Koul, 2020). Bt is a naturally occurring Gram-positive bacterium that resides in the soil. One of the remarkable characteristics of Bt is its ability to produce insecticidal proteins, known as delta endotoxins (δ-endotoxins) or Cry pesticidal proteins. These proteins are highly effective against a wide range of targeted insect pests, including lepidopteran pests. Over 1,100 of them have been identified and classified based on their structure, sequence homology, and activity (Crickmore et al., 2023). They often target specific insect species within the same taxonomic family or order, possessing a relatively narrow activity spectrum (Schnepf et al., 1998; Crickmore et al., 2021). For decades, Bt spores have been widely used as a biological pesticide safe for humans, animals, non-target invertebrates, and the environment. In parallel, more than 300 genetically engineered events in various crops (maize, cotton, soybean, rice, eggplant, sugarcane, tomato, cowpea, and poplar) expressing Bt proteins have received regulatory approvals (ISAAA, 2023).

According to the Food and Agriculture Organization of the United Nations, eucalyptus farms cover approximately 0.5% (∼22.57 million hectares) of the world’s forested areas (FAO, 2020) but fulfill approximately 10% of the current global demand for roundwood. Its significant contribution to wood supply renders eucalyptus a crucial species for present and future wood production. This helps in protecting native forests by reducing the utilization of wood from these ecologically important areas. Brazilian eucalyptus farms are managed using modern agricultural practices akin to other row crops, and like any other crop, they are challenged by pests, including exotic pests introduced by eucalyptus originating from Australia and native Brazilian pests that have rapidly adapted to a eucalyptus diet (Paine et al., 2011; Mota et al., 2022).

Brazilian eucalyptus farms typically experience sporadic instances of lepidopteran pest infestations. However, there is a significant likelihood of a major outbreak to occur during the 6–7 years of the rotation cycle. Even a single infestation of defoliators can inflict substantial damage, estimated to cause a loss of 13%–40% in the annual yield (Barbosa et al., 2022), which translates to a reduction of 9%–19% in the wood volume during harvest. Additionally, when the tree’s defense mechanism is activated, it leads to undesirable changes in the wood’s properties and an increase in the lignin content. Consequently, these cumulative effects result in a decline in pulp production by 15%–24% (Zanuncio et al., 2020).

In Brazil, the brown looper moth *Thyrinteina arnobia* (Stoll, 1782) (Lepidoptera: Geometridae) is a major lepidopteran pest of eucalyptus trees. Originally infesting native Myrtaceae hosts, like guava (*Psidium guajava*) (Holtz et al., 2003; Barbosa et al., 2022), *T. arnobia* has expanded its range to include eucalyptus. Since 2015, over 413,000 hectares of eucalyptus fields in Suzano S.A. have been reported to being infested by *T. arnobia*. The two main control methods involve releasing pupal parasitoid wasps, such as *Tetrastichus howardi* (Olliff, 1893) and *Trichospilus diatraeae* (Cherian and Margabandhu, 1942) (Hymenoptera: Eulophidae) (Barbosa, et al., 2022), and applying Bt biopesticides, such as DiPel^®^. Bt biopesticide applications are carried out after the manual monitoring and detection of caterpillars and their damage in the field (McDowell and Mann, 1991). However, this treatment does not fully prevent damage, and each biopesticide application increases the environmental footprint. Therefore, additional control strategies are needed to protect eucalyptus farms better.

As part of a sustainable, eco-friendly initiative, FuturaGene, the Biotech Division of Suzano S.A., has developed the insect-resistant genetically modified eucalyptus event 1521K059, expressing three Bt Cry pesticidal proteins, Cry1Ab, Cry2Aa (also in DiPel^®^), and Cry1Bb, targeting *T. arnobia*, as well as the selectable marker kanamycin resistance gene *nptII* (Beck, et al., 1982; Toth, et al., 2007). The three pesticidal protein genes and the selectable marker were cloned adjacent to each other on a single-transfer DNA (T-DNA), resulting in a single genomic insertion site after the transformation using *Agrobacterium tumefaciens* (Prakash and Gurumurthi, 2009).

Under the Brazilian legislation, Normative Resolution 32 (CTNBIO, 2021) governs the standards for the commercial release and monitoring of genetically modified organisms. This resolution does not specify the required or excluded studies, and it does not differentiate between the processes associated with the events intended for human or animal consumption. The responsibility lies with the petitioner to generate scientific data, demonstrating to the National Technical Commission on Biosafety (CTNBio) that the specific genetically modified organism (GMO) event in question poses no risks to the environment, human health, or animal health. Typically, studies are conducted based on the precautionary principle, often following the precedents of the previously approved submissions in Brazil and globally. Brazil’s regulatory system is considered one of the most robust worldwide, having conducted risk assessment evaluations for over 25 years without detecting any adverse effects from commercially approved GMOs.

After thorough biosafety assessments in the laboratory and field for over 2 years, the event 1521K059 was approved by the CTNBio for commercial use in Brazil. This manuscript presents the key findings from the extensive biosafety evaluations of the eucalyptus event 1521K059 in the field and laboratory safety tests of transgenically expressed Cry1Ab, Cry1Bb, and Cry2Aa pesticidal proteins. The safety assessments of the NPTII protein were published before (Fuchs et al., 1993; Avisar et al., 2023). Although Cry1Ab has a well-established history of biosafety studies in other commercial GM crops (Federici and Siegel, 2008; Wolt et al., 2008; ILSI, 2011), we present the accumulated data for this pesticidal protein alongside Cry2Aa and Cry1Bb. Cry2Aa and Cry1Bb pesticidal proteins have limited biosafety data in the literature and worldwide regulatory applications since they have been rarely used commercially thus far (AgbioInvestor, 2023). Furthermore, this work emphasizes the potential of insect-resistant eucalyptus as a new tool in pest management and in the promotion of sustainable practices and environmentally friendly solutions in the tree crop sector.

## Materials and methods

### Genomic insertion site identification

Genomic DNA was extracted from 1521K059 IR GM eucalyptus using the cetyltrimethylammonium bromide (CTAB) protocol (Richards et al., 1994). Fresh leaf tissue (2 g) was frozen in liquid nitrogen and finely powdered. Subsequently, 15 mL of the extraction buffer (2% CTAB, 100 mM Tris at pH 8, 1.5 M NaCl, 0.2 mM EDTA at pH 8, 1% β-mercaptoethanol, and 0.1% PVP) was added. The mixture was then incubated at 65°C for 60 min, periodically swirled, and then cooled to room temperature. Next, the sample was thoroughly mixed with 15 mL chloroform–isoamylic alcohol and then centrifuged at 10,000 *g* for 15 min at 22°C. Then, the supernatant was carefully transferred to a new tube, and the chloroform isoamylic alcohol step was repeated twice. Then, an equal volume of ice-cold isopropyl alcohol was added, and the tube was incubated at −20°C for 30 min. The samples were then centrifuged at 20,000 *g* for 20 min at 4°C. The resulting supernatant was discarded, and the pellet was resuspended in 500 μL of 70% ice-cold ethanol and then centrifuged at 20,000 *g* for 2 min at 4°C. After the aspiration of 70% ethanol, the tubes were left open at room temperature to allow complete ethanol evaporation. The pellet was then resuspended in 250 μL RNase (10 ng/μL; Sigma R6513) in the Tris–EDTA buffer and stored at 37°C until complete dissolution of the pellet (final DNA concentration of 20 ng/μL).

The extracted DNA (0.5 μg) was sequenced on the Illumina HiSeq 2500 platform, utilizing a single individual lane, which generated raw read data (150PE, 80 gigabytes). Read mapping was performed using Geneious Prime software version 11 (http://www.geneious.com). Reads that successfully aligned with both T-DNA and genome DNA sequences were used to determine the specific location of the insert within the genome. The insertion within the genome was located using a published eucalyptus genome reference, BRASUZ 2.0 (Myburg et al., 2014).

### Bioassays with *Thyrinteina arnobia*

A laboratory population of *T. arnobia* was maintained in a temperature-controlled room (25°C ± 2°C), with a relative humidity of 70% ± 10% and a 12-h light phase, as described by Oliveira et al. (2005). For the bioassays, the natural leaf diet was replaced with an artificial diet. To prepare one batch of the diet, the following steps were followed: boiling water (544 mL) was combined with 12.5 g of wheat germ (Jasmine, Campina Grande do Sul, PR, Brazil), 9 g of the yeast extract (local market, purchased by kilogram without brand definition), 67.3 g of white corn flour (produced in the laboratory, white corn from Embrapa, Brazilian Enterprise of Agriculture and Livestock Farming Research), 25.5 g of the soybean meal (Ecobio, Coronel Bicaco, RS, Brazil), 5.3 mL of soy oil (Cargill, Uberlândia, MG, Brazil), and 5.3 g of skimmed milk (La Serenissima, Barueri, SP, Brazil). The mixture was stirred for 10 min. Water (300 mL) and agar (12.5 g) (PhytoTech Labs, Lenexa, KS, USA A296) were boiled and then mixed with the above mixture. The mixture was allowed to cool to 45°C, and the Vanderzant vitamin mixture for insects (0.5 mL) (Sigma-Aldrich, Darmstadt, HE, Germany V1007), Nipagin (1.35 g) (Sigma-Aldrich, Darmstadt, HE, Germany H5501), sorbic acid (0.68 g) (Sigma-Aldrich, Darmstadt, HE, Germany S1626), ascorbic acid (3.6 g) (Sigma-Aldrich, Darmstadt, HE, Germany A4544), Wesson’s salt (2 g) (Lab House, Belo Horizonte, MG, Brazil 16,632), V8™ tomato and vegetable juice (50 mL) (Campbell Soup Company, Camden, NJ, USA), and any substances being tested were added and mixed. The diet was dispensed into testing plates or tubes for immediate use.

To assess the individual activity of each pesticidal protein, the *Cry1Ab*, *Cry1Bb*, and *Cry2Aa* genes were individually cloned between the *cauliflower mosaic virus* (CaMV) 35S promoter and the T-Nos terminator. These genetic constructs were then introduced into eucalyptus plants using the *A. tumefaciens* transformation method (Prakash and Gurumurthi, 2009). The bioassays involving single pesticidal protein Cry1Ab or Cry1Bb, or Cry2Aa-expressing eucalyptus and the wild-type (wt) FGN-K, were conducted using 3-month-old plantlets. These plantlets were approximately 60 cm tall and had approximately 10 leaves each. They were produced from cuttings from a polycarbonate greenhouse equipped with a pad-fan cooling system. The greenhouse-maintained temperatures ranged from 18°C to 28°C, and the relative humidity was between 70% and 90%.

The bioassays were conducted in a temperature-controlled room (25°C ± 2°C), with a relative humidity of 70% ± 10% and a 12-h light phase. For each experiment, 10 second-instar *T. arnobia* caterpillars were placed on each plantlet (five replicates per treatment). Each plantlet was carefully placed between two 1-L clear polypropylene deli containers, which were then securely taped together. The upper lid of the container was swapped with a 50-mesh net. A caterpillar’s survival was assessed after a period of 6–7 days (Tukey’s test was employed at a 1% significance level to enhance the sensitivity of the test in detecting differences).

The evaluation of event 1521K059 plantlets (Supplemental Figure S8) was conducted within 50-mesh-net cages under the same controlled conditions, as mentioned above. In each cage, 10 second-instar *T. arnobia* caterpillars were introduced, and both mortality and leaf damage were documented after 6 days. Field bioassays with *T. arnobia* were conducted in separate locations from the regulatory field trials mentioned below to avoid any potential harm to the trees of the regulatory trials.

The experiments were carried out in two farms in São Paulo and one farm in Mato Grosso do Sul. The test on each site involved 6-month-old trees arranged in five linear blocks, with each block containing six trees. The spacing between the trees and rows was 3.0 m. The bioassays included event 1521K059, which expresses all three pesticidal proteins and the FGN-K wild-type clone. Insect-proof cages made of mesh bags were used, and each cage was placed on a branch. A total of 30 neonate caterpillars, hatched on the same day, were introduced into each cage. The branches were thoroughly inspected and cleaned before the release of the caterpillars to ensure the absence of predators inside the cages. The cage bases were securely sealed with cord and tape, to prevent the entry or exit of insects. After a period of 7 days, the cages were opened and the number of surviving caterpillars was counted (Tukey’s test at the 1% significance level).

Laboratory bioassays were performed using mature leaves collected from a designated field site in the State of São Paulo (SP). The fresh leaves which were not diluted or mixed with any other part of the diet, served as the undiluted control sample. These leaves were then lyophilized and diluted in the diet. For each dilution, the ratio of the weight of lyophilized leaves to their original fresh weight was multiplied by 150 (the final volume of the diet in ml) and divided by the dilution factor. This calculation determined the quantity of lyophilized leaves in grams to be combined with 150 mL of the artificial diet. Dilutions of 25, 50, 100, 200, 400, 800, 1,200, 1,600, and 2,000 of leaves from both the event 1521K059 and the wild-type eucalyptus FGN-K were prepared. Each dilution was tested on 20 microtubes, each containing one *T. arnobia* neonate caterpillar. After 7 days of exclusive feeding on the diet containing diluted leaves, the surviving caterpillars were transferred to the original growth container (Oliveira et al. (2005) and were provided with a food source devoid of pesticidal proteins. They were allowed to develop and complete their life cycle until reaching adulthood. A qualitative analysis in each group was employed to document the highest life stage attained by individuals for each dilution, without statistical tests.

### *In silico* allergenicity and toxicity analyses

Allergenicity and toxicity analyses are the integral components of all biosafety assessment studies submitted in Brazil, as mandated by the FAO guidelines for any commercially planted GM crop (Hautea, 2009). To assess the potential allergenicity of Cry1Ab, Cry1Bb, and Cry2Aa, their amino acid sequences (Supplemental Figure S1) were analyzed using the COMPARE allergen database (van Ree et al., 2021). The known allergen profilin (GenBank: AGA84056.1) was used as a positive control. Three different types of sequence comparisons were performed using the FASTA search (Pearson, 1990) to identify any similarities between known allergenic proteins. The first comparison involved searching for similarities in the full-length sequence, with a specific emphasis on detecting highly distinctive resemblances that cannot be attributed to a random chance. In protein alignment searches, the accepted threshold for a random chance is less than 1 in 1,000, denoted by a parameter called the E-value that should be lower than 10e-4 (0.001) (Karlin and Altschul, 1990). The second comparison utilized an 80-mer sliding window search to identify instances where the identity exceeded 35%. Finally, an 8-mer sliding window search was conducted to identify any peptides of the complete identity.

The BLASTP tool (version: 2.11.1+), a program that finds protein sequences similar to a given target sequence, was utilized (Altschul et al., 1997) to assess the potential toxicity of Cry1Ab, Cry1Bb, and Cry2Aa in humans and animals. The known human toxin ricin (UniProt P02879) was used as a positive control. The NCBI nr database was employed for this purpose, using the following configuration settings: max target sequence = 5,000; E-value threshold = 0.001; word size = 6; matrix = BLOSUM62; gap costs = existence: 11; extension: 1; filter for low complexity = off. Moreover, a search was conducted on the UniProt database using the BLASTP tool, using the following configuration parameters: target database = UniProtKB reference proteome plus Swiss-Prot; E-value threshold = 0.001; matrix = BLOSUM62; filter for low complexity = off; gap penalty = yes; hits = 1,000. In both instances, the search outcomes were screened for the presence of the terms “toxic,” “toxin,” “anti-nutrition,” “agglutinin,” “trypsin inhibitor,” and “protease inhibitor” in their descriptions. Additionally, the Toxic Exposome Database (T3DB) was employed to identify any homology to the known toxins (Lim et al., 2010; Wishart et al., 2015). The homology search was conducted using the BLASTP tool with the following configuration parameters: cost to open a gap = −1, cost to extend a gap = −1, penalty for the mismatch = −3, reward for the match = 1, and expectation value = 0.00001.

### Recombinant proteins

Recombinant Cry1Ab, Cry1Bb, and Cry2Aa pesticidal proteins (>5 g each) were produced as needed in *Pseudomonas fluorescens* bacteria, by Fraunhofer-Gesellschaft (Fraunhofer-Gesellschaft, Munich, BY, Germany), and analyzed by Schafer Scientific Solutions LLC (Schafer Scientific Solutions LLC, Carmel, IN, USA). In order to stabilize the proteins, the potential trypsin cleavage site at the N-terminal was eliminated by introducing mutations (R28del + I29Q for Cry1Ab and R5Q + R34Q for Cry1Bb). For Cry2Aa, a His6 tag was attached at the C-terminal to simplify purification in a nickel column. These modifications are located outside the active domains of the pesticidal proteins (Sanahuja et al., 2011), indicating that their impact on the protein activities should be minimal. The inclusion bodies containing these proteins were collected from cells that were grown in a standard 50L fermentation process for 48 h. Trypsin was used to activate Cry1Ab and Cry1Bb pesticidal proteins by the cleavage of the C-terminal site. The purity levels (protein/protein) and concentrations (% of the active ingredient per powder mass) were as follows: Cry1Ab purity was greater than 80% with a concentration of 28%; Cry1Bb purity was greater than 35% with a concentration of 20%; Cry2Aa purity was greater than 90% with a concentration of 20%. The activity of the purified proteins was validated by the manufacturers using *Helicoverpa armigera* (Lepidoptera: Noctuidae) larvae for Cry1Ab, *Manduca sexta* (Lepidoptera: Sphingidae) for Cry1Bb, and *Spodoptera exigua* (Lepidoptera: Noctuidae) for Cry2Aa. The immunoreactivity of the proteins was assessed using enzyme-linked immunosorbent assay (ELISA); Cry1Ab was assessed using the Agdia ELISA Kit PSP 06200 (Agdia, Inc. IN, USA), Cry2Aa was assessed using the Agdia ELISA Kit PSP 05801 (Agdia, Inc. IN, USA), and Cry1Bb was assessed using the Eurofins-Abraxis ELISA Kit PN 599100 (Eurofins Abraxis, Warminster, PA, USA).

### Simulated gastric fluid and simulated intestinal fluid digestibility

The digestibility assays were conducted as an additional safety measure based on a tiered approach (Garcia-Alonso et al., 2006). The objective was to demonstrate human safety under any worst-case scenario involving the ingestion of plant parts or pollen. Protein susceptibility to degradation by digestive enzymes was evaluated in the simulated gastric fluid (SGF) and the simulated intestinal fluid (SIF). The study followed the protocols described by Thomas et al. (2004) and Fu et al. (2002). Cry1Ab, Cry1Bb, and Cry2Aa (0.1 mg/mL each), along with bovine serum albumin (BSA, 0.247 mg/mL) [Sigma-Aldrich, Darmstadt, HE, Germany cat#: A7638] and β-lactoglobulin (β-lac, 0.272 mg/mL) [Sigma-Aldrich, Darmstadt, HE, Germany cat#: L7880], were incubated with SGF or SIF at 37°C for varying intervals of time. For SGF, the digestion intervals were approximately 30 s and 1, 2, 4, 8, 16, and 32 min. For the SIF, the digestion intervals were approximately 30 s; 1, 2, 4, 8, 16, and 32 min; and 1, 2, 4, 24, and 48 h. Proteins were analyzed by SDS-PAGE, with gels stained with SimplyBlue SafeStain (Invitrogen, Frederick MD, USA, LC6060) for approximately 1 h and then destained in ultrapure water with agitation at 50 rpm for approximately 16 h. Gel images were captured and analyzed using an iBright 1500 system (Thermo Fisher Scientific Inc. MA, USA) using iBright Analysis Software (version 4.0.0).

### Thermostability assessment

Thermostability tests were conducted with the aim of demonstrating that, during the heat stage of pulp production (>140°C; Tran, 2002), the transgene protein products undergo degradation. The thermal stability of Cry1Ab, Cry1Bb, and Cry2Aa was evaluated by subjecting the proteins (200 ng/μL) to increasing temperatures (20, 40, 60, 80, 90, 100, 110, and 120°C) for 20 min, in 50 mM of the CAPS buffer, and pH 10.0. Following the heating process, the samples were centrifuged at 20,000 *g*, 4°C, for 2 min. The soluble portions were subsequently subjected to SDS-PAGE and ELISA, as described above. Gel images and densitometry analyses were captured and processed using Bio-Rad’s Image Lab software version 6.1 (Bio-Rad Laboratories, Inc. CA, USA).

### Regulatory field trials

The eucalyptus event 1521K059 and the wt clone FGN-K were planted at four sites in Brazil: two in the State of São Paulo, one in the State of Bahia (BA), and one in the State of Maranhão (MA). The planting design consisted of square plots, each containing 16 plants. Five square plots of each clone/event were randomly distributed in blocks within the field, alongside other plots of unrelated clones that were not part of the experiment (Supplemental Figure S2).

### Pesticidal protein expression levels

Tissue samples of young and mature leaves, stems, roots, flower buds, and pollen were collected from 6-, 12-, and 24-month-old eucalyptus event 1521K059 trees across the four regulatory trial farms. The tissues were ground in liquid nitrogen using a Thermo Scientific TissueLyser II and then lyophilized for 96 h using a Labconco FreeZone 1 L Benchtop Freeze Dry System (Labconco Corporation, MO, USA) set to −56°C. Cry1Ab, Cry1Bb, and Cry2Aa concentrations were determined using the ELISA kits listed above, following the manufacturers’ protocol. The recombinant proteins described above were used for the standard curves. The highest pesticidal protein concentrations found were used in the margin of exposure calculations.

### Margin of exposure calculations for non-target indicator species

Exposure studies were conducted on honey bee *Apis mellifera* (Hymenoptera: Apidae) (larvae and adults), earthworm *Eisenia fetida* (Opisthopora: Lumbricidae), springtail *Folsomia candida* (Collembola: Isotomidae), and aquatic invertebrate *Daphnia magna* (Anomopoda: Daphniidae), following OECD protocols 239, 245, 222, 202, and 232, respectively (OECD, 2004a; OECD, 2004b; OECD, 2016; OECD, 2017; OECD, 2021). The diet, growth conditions, and experimental procedures’ result analyses and statistics were as described in the protocols.

The no-observed-effect concentration (NOEC) for each organism or the equivalent no-observed-effect dose (NOED) in the case of *A. mellifera* was the highest soluble recombinant Cry1Ab, Cry1Bb, or Cry2Aa pesticidal protein concentration (one high concentration per species) that was incorporated into the diet or liquid habitat (for *D. magna*), according to the OECD protocols, for each indicator species.

The estimated environmental concentrations (EECs) of pesticidal proteins for earthworm *E. fetida* and springtail *F. candida* were the highest measured concentrations (in μg/g) of each protein found in the tissues of the event 1521K059 (Table 4).

**FIGURE 1:**
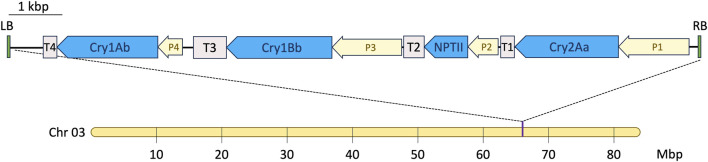
T-DNA insertion site in the transgenic eucalyptus event 1521K059. A single insertion was identified on chromosome 3 at approximately 66 Mbp. P1—*cauliflower mosaic virus* (CaMV) 35S promoter fused to the eucalyptus translation elongation factor EF-1 alpha intron (Eucgr J01112); P2—CaMV 35S promoter; P3—eucalyptus ribulose bisphosphate carboxylase small subunit promoter (Eucgr J01502); P4—CaMV 35S promoter; T1 and T4—*Agrobacterium tumefaciens* nopaline synthase terminator; T2—CaMV 35S terminator; and T3—eucalyptus ribulose bisphosphate carboxylase small subunit terminator (Eucgr J01502).

To calculate the estimated environmental dose (EED) for *A. mellifera*, the maximum pollen intake per larval development stage of 2.04 mg/pollen (Babendreier et al., 2004) and the maximum daily pollen intake for adult worker bees of 4.3 mg/pollen (Crailsheim et al., 1992) were each multiplied by the highest measured concentration of each pesticidal protein found in pollen from the event 1521K059. Since the Cry1Bb protein is regulated by a promoter specific to green plant tissues (Figure 1), it was not detected in the pollen of the event 1521K059. Therefore, the limit of quantification (LOQ) of 1.24 μg/g, stated in the Eurofins Abraxis detection ELISA kit (PN 599100), was used as a conservative estimate of the maximum Cry1Bb concentration in 1521K059 pollen.

To conservatively estimate the EEC for *D. magna*, a model was used that assumed the living tissue biomass from 10 ha of transgenic eucalyptus drains into a 20,000-cubic meter pond (Carstens et al., 2012). The amount of the living tissue biomass per hectare of eucalyptus was set to 25,200 kg based on published data (Ludvichak et al., 2022). The EEC was calculated by taking 10 ha of the biomass at 25,200 kg/ha, multiplying it by the maximum measured concentration of each protein in the tissues of the event 1521K059, and dividing it by the 20 million-liter pond volume.

Margin of exposure (MoE) values were calculated for each of the four model non-target organisms and each protein using the ratio NOEC/EEC or NOED/EED, respectively.

### Soil microbial community analysis

Twenty four months after planting, microbial diversity and density studies were conducted across the four regulatory trial farms, following the methods outlined by Avisar et al. (2023). In summary, soil samples were obtained from each of the event 1521K059 and FGN-K plots (five per plot) in all four field trials (five plots per field), using a clean auger, reaching a depth of 15 cm. Prior to collection, the sampling locations were carefully cleared of any weed and plant remnants. An amount of 1.0 g of the soil was diluted in 10 mL of phosphate-buffered saline, comprising 137 mM NaCl, 2.7 mM KCl, 10 mM Na_2_HPO_4_, and 1.8 mM KH_2_PO_4_. The mixture was thoroughly stirred and then centrifuged at 1000 *g* for 5 min. Subsequently, 100 µL of the resulting solution was inoculated into the appropriate culture medium, as outlined in Avisar et al. (2023). Microbial density was expressed as the logarithm (base 10), with colony-forming units (CFUs) per gram of soil. To evaluate microbial diversity, ribosomal RNA sequencing was employed, and the analysis utilized the “alpha diversity” tool from QIIME software (Caporaso et al., 2010). The principal coordinate analysis (PCoA) was conducted to compare sample groups based on the phylogenetic and count-based distance metrics (Hammer et al., 2001; Podani and Miklós, 2002).

### Arthropod collection and analysis

Arthropods were collected and examined by following the methods outlined by Avisar et al. (2023). In summary, in all four regulatory trial farms, a total of five distinct sampling techniques were utilized at various points during the plant growth cycle, when the plants were approximately 4, 10, 12, 19, and 23 months old. These methods included the following (Supplemental Figure S3): modified “beating sheet/net,” where the branches were vigorously shaken for 30 s inside a plastic bag (10 samples per plot, at only 4 years and 10 months of age before the trees were too high to shake the branches); pitfall traps: traps measuring 10 cm in diameter and 15 cm in height were placed at the center of each plot, filled with a solution (1%–2% detergent and 4% formaldehyde), and left for 72 h; adhesive cards: attractive yellow adhesive sheets (14 × 23 cm, ISCA brand) were positioned at the center of each plot, at the height of the treetops, and left for 72 h; soil collection: the samples (10 samples per plot, which are 10 cm in diameter and 5 cm in depth) were obtained, and the species were retrieved using the Berlese–Tüllgren funnel method (Brown, 1973); litter collection: samples (five samples per plot, 25 cm^2^) were obtained, and the species were retrieved using the Winkler extractor method (Besuchet et al., 1987; Sabu et al., 2011).

Following the collection, the samples were preserved in a solution of 70% ethanol and 5% glycerin. They were then classified into different taxa by comparing them with reference collections or with the literature. The “Total” number of observed arthropods, “Richness” (defined as the number of observed species), and “Diversity” (defined as the inverse of the sum of the squares of the observed numbers of each species, divided by the total number of observed species) were analyzed using ANOVA with the agricolae package (version 1.3.5) in the R programming language (version 4.3.0). The significance level was set at alpha = 0.05. Statistical comparisons were carried out between the control group (FGN-K) and the eucalyptus event 1521K059. To control for the false discovery rate (FDR; type 1 statistical error), adjustments were made upward based on the magnitude of the F-test. Eta-squared effect sizes (ɳ^*2*^) were calculated for every ANOVA using the *eta_squared*() function from the “effectsize v 0.8.6” R package (Ben-Shachar et al., 2020).

### Organic material degradability assays

Screened nylon bags with a mesh size of 2 mm and dimensions of 20 × 20 cm were used to contain the litter samples. Each bag was filled with 35 g of biomass, comprising 5 g of branches and 30 g leaves collected right at the regulatory trial sites. At each of the four trial sites, five bags containing either the biomass from the event 1521K059 or commercial reference eucalyptus FGN-K were placed touching the ground in each plot. Altogether, there were five bags per sample type per time point at each location. On both days 0 and 180, the samples were analyzed following the methodology and calculations described by Santos and Whitford (1981). Initially, the samples were dried for approximately 1 h, at a temperature of 60°C–70°C, and the weight of the dry matter was recorded. Then, the samples were incinerated in a muffle furnace at 700°C to determine the content of ashes and organic matter. The loss of ashes and organic matter dry weight was calculated by comparing the results to those obtained on day 0. The average values were calculated across all sites for both the event 1521K059 and the commercial reference. Analysis of variance was conducted, and mean separations within the treatment and weight were determined by Tukey’s test at a 5% error probability.

## Results

### Identification of the genomic insertion site

The insertion site of the T-DNA encoding the triplet pesticidal Cry proteins was identified by deep DNA sequencing and genome read mapping. Based on the publicly available eucalyptus genome, BRASUZ 2.0 (Myburg et al., 2014), it was established that event 1521K059 possesses a single heterozygous insertion site. This insertion site was identified on one of the two chromosomes #3 of the event 1521K059, located at approximately 66 Mbp, while no endogenous gene was affected by the insertion. Figure 1 illustrates a single inverted insertion detected in the genome. The complete sequencing of all four expression cassettes, starting from the T-DNA right border, identified the *Cry2Aa* gene controlled by the *cauliflower mosaic virus* 35S promoter, which was fused to the eucalyptus translation elongation factor, the EF-1 alpha gene (Eucgr J01112) intron, and to the *A. tumefaciens* nopaline synthase terminator (T-Nos). In addition, it identified the *nptII* selectable marker gene under the control of the CaMV 35S promoter and terminator, the *Cry1Bb* gene under the eucalyptus ribulose-bisphosphate carboxylase small subunit (Eucgr J01502) promoter and terminator, and, finally, the *Cry1Ab* gene controlled by the CaMV 35S promoter and terminated by T-Nos.

### Event 1521K059 can effectively control the *T. arnobia* pest

**FIGURE 2:**
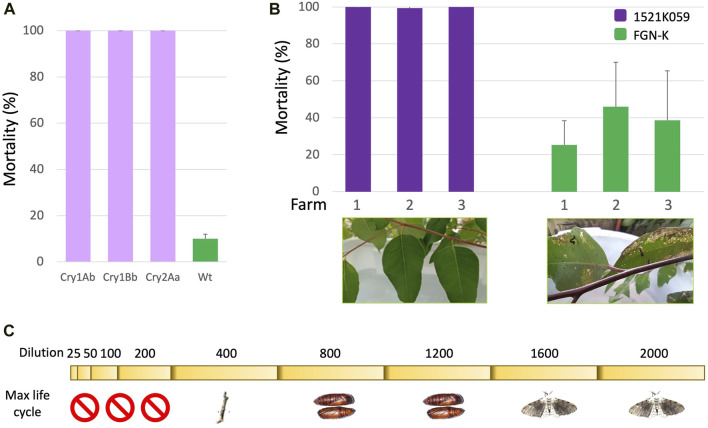
Analyses of transgenic eucalyptus expressing pesticidal proteins. **(A)** Eucalyptus events expressing Cry1Ab, Cry1Bb, or Cry2Aa effectively controlled second-instar *T. arnobia* caterpillars, resulting in 100% mortality within 7 days (Tukey’s test at the 1% significance level). **(B)** In-field trials (two farms in São Paulo, SP-1 and SP-2, and one farm in Mato Grosso do Sul, MS) using branch cages; the event 1521K059 achieved 99.3%–100% mortality of *T. arnobia* neonate caterpillars, compared to the 20%–40% mortality in wild-type branch cages after 7 days of exposure. The representative leaf damage shown below in the graphs demonstrates the protection provided by the event 1521K059 against *T. arnobia* caterpillars (Tukey’s test at the 1% significance level). **(C)** Dilutions of the event 1521K059 leaf extracts in the *T. arnobia* diet in laboratory assays. The 200-fold dilution was sufficient to eliminate all neonate caterpillars. The 400-fold dilution arrested the life cycle of the surviving caterpillars before the pupal stage. The 1,200-fold dilution still prevented adult emergence. Higher dilutions allowed caterpillars to complete the life cycle to adulthood.

Field and laboratory evaluations were conducted to assess the efficacy of the IR GM eucalyptus event 1521K059 against *T. arnobia*. Initially, we conducted tests to determine whether each individual pesticidal protein, expressed in eucalyptus, can effectively control the target pest on its own. Single pesticidal protein Cry1Ab or Cry1Bb, or Cry2Aa-expressing eucalyptus and WT FGN-K were employed to evaluate the activity of each pesticidal protein. The GM eucalyptus events expressing single pesticidal proteins effectively controlled second-instar *T. arnobia* caterpillars, resulting in 100% mortality within 7 days (Figure 2A).

Due to a low chance of a natural infestation, the efficacy of the event 1521K059 in the field was tested by intentional infestation in branch cages. The three field trials indicated that event 1521K059’s branches conferred a mortality rate of 99.3%–100% for *T. arnobia* caterpillars (Figure 2B). In comparison, the mortality rate of the neonate caterpillars exposed to wt FGN-K branches was 20%–40% (Supplemental Figure S8), further substantiating the efficacy of event 1521K059 cuttings in combating *T. arnobia* second-instar infestation and preventing any damage within a laboratory cage environment.

When testing the impact of diluted leaf extracts from event 1521K059 (Figure 2C) in laboratory tests, even a 200-fold dilution of the extracts was sufficient to eliminate all the feeding neonate *T. arnobia* caterpillars. When exposed to a 400-fold dilution, some caterpillars survived but exhibited abnormal growth by failing to progress to the pupal stage, when reintroduced to their normal diet. Caterpillars exposed to event 1521K059’s leaves diluted up to 1,200 times were able to enter the pupal phase but failed to emerge as adults. However, from a dilution of 1,600-fold and above, the caterpillars completed their development and successfully emerged as adults. *T. arnobia* caterpillars were fed undiluted wt leaves, as well as all dilutions of the diet, completed their entire life cycle, and developed into adults.

### *In silico* allergenicity and toxicity analyses

*In silico* analyses performed to assess the potential allergenicity of Cry1Ab, Cry1Bb, and Cry2Aa, based on datasets as of 2022, found no significant matches (Table 1), indicating that these proteins, like other Bt Cry pesticidal proteins (Randhawa et al., 2011), are non-allergenic. The full-length alignments demonstrated no noteworthy similarity to any known allergen, and all results had E-values above 10e-4, suggesting a lack of meaningful biological resemblance between the sequences (Pearson, 1999). Furthermore, when employing a sliding window of 80 amino acids, no relevant alignments with an identity greater than 35% were observed for any of the tested proteins. Additionally, an analysis of eight amino acid peptides found no matches. The positive control profilin had more than 100 hits in all the three tests.

**TABLE 1:** *In silico* allergenicity and toxicity analyses.

		Allergenicity			Toxicity				
Protein	Size (aa)	Full-length	80-mer	8-mer	NCBI nr		UniProt		T3DB
		Hit	Hit	Hit	Alignment	Toxic term	Alignment	Toxic term	Hit
Cry2Aa	633	0	0	0	1,781	0	115	0	0
Cry1Bb	655	0	0	0	2,094	0	150	0	0
Cry1Ab	622	0	0	0	2,073	0	135	0	0
Positive control									
Profilin	131	>100	139	138					
Ricin	576				8,102	>90%	>1,000	>90%	1

Searches conducted in the NCBI nr and UniProt databases using the Cry1Ab, Cry1Bb, and Cry2Aa protein sequences revealed alignments with other Cry pesticidal proteins, as well as proteins from the “δ-Endotoxins” and “Endotoxin_N domain-containing protein” groups. Some putative proteins showing a partial similarity to conserved domains found in Cry pesticidal proteins were also identified. However, apart from these, no relevant occurrences of the terms “toxic,” “toxin,” “anti-nutrition,” “agglutinin,” “trypsin inhibitor,” and “protease inhibitor” were found in the BLASTP output files (Table 1). The positive control ricin had thousands of known similarities with toxins and is registered in the T3DB.

### Simulated gastric fluid and simulated intestinal fluid digestibility

In the digestibility assays, Cry1Ab, Cry1Bb, and Cry2Aa exhibited similar behaviors to the control BSA in the SGF and SIF, i.e., rapidly digested (30 s–4 min) in the SGF but resistant to digestion (up to 48 h) in SIF (Table 2; Supplementary Figure S4; Supplementary Figure S5). Conversely, β-lactoglobulin showed high digestibility in the SIF but resisted digestion in the SGF.

**TABLE 2:** Digestibility results in the simulated gastric fluid (SGF) and simulated intestinal fluid (SIF).

Treatment	Protein	Maximum detection time
SGF	β-lac	32 min
	BSA	30 s
	Cry1Ab	4 min
	Cry1Bb	2 min
	Cry2Aa	30 s
SIF	β-lac	30 s
	BSA	48 h
	Cry1Ab	48 h
	Cry1Bb	48 h
	Cry2Aa	2 h

### Thermostability assessment

In thermostability studies, Cry1Ab and Cry2Aa were degraded as the temperatures were increased. The degradation was particularly pronounced at temperatures above 40°C (Table 3; Supplementary Figure S6). Cry1Bb underwent significant degradation, primarily at temperatures exceeding 60°C. The solubility of the pesticidal proteins gradually decreased as the temperature increased, as determined by densitometry. The immunoreactivity of the proteins, assessed by reduced binding to ELISA plates, also showed a gradual decrease with the increasing temperatures. At temperatures above 60°C, the antibodies in the ELISA plates failed to recognize the proteins and immunoreactivity reached 0%, indicating a substantial loss of conformation at higher temperatures (Table 3).

**TABLE 3:** Densitometric measurement (by SDS-PAGE) and immunoreactivity (by ELISA) of the effect of heat treatment on pesticidal proteins after 20 min of exposure.

Treatment (°C)	Densitometry (%)			Immunoreactivity (%)		
	Cry1Ab	Cry1Bb	Cry2Aa	Cry1Ab	Cry1Bb	Cry2Aa
0	100	100	100	100	100	100
20	100	100	100	100	100	99
40	100	100	100	100	95	81
60	54	100	83	5	93	37
80	53	38	0	0	0	0
90	33	29	0	0	0	0
100	5	23	0	0	0	0
110	1	12	0	0	0	0
120	1	1	0	0	0	0

### Maximum concentration levels

The biosafety tests and estimates of environmental exposure are based on the maximum recorded levels of the expression and concentration of pesticidal proteins in the evaluated event 1521K059. The highest concentration values across four farms and trees aged 6–24 months were used for the margin of exposure calculations. These were 53.76 μg/g for Cry1Ab, 8.33 μg/g for Cry1Bb, and 9.73 μg/g for Cry2Aa in leaf tissues. In pollen, the levels were 5.08 μg/g for Cry1Ab and 1.53 μg/g for Cry2Aa. The Cry1Bb expression is limited to green tissue by the Eucgr J01502 promoter and terminator (Figure 1), so no expression was detected in the stem, roots, and pollen. Therefore, the limit of quantification value of 1.24 μg/g for Cry1Bb was used as described in the methods.

### Pesticidal protein margin of exposure for non-target indicator species

To evaluate the risk to non-target organisms upon their potential exposure to the pesticidal proteins in the eucalyptus event 1521K059, the worst-case scenario estimated EEC or EED of each protein was compared to the NOEC or NOED of the representative indicator species. The highest recorded Cry1Ab, Cry1Bb, and Cry2Aa concentrations (Table 4) were used to calculate the EEC or EED representing worst-case exposures. For earthworm and springtail, the EEC was the maximum leaf concentrations in their diet: 53.76 μg/g for Cry1Ab, 8.33 μg/g for Cry1Bb, and 9.73 μg/g for Cry2Aa. For *D. magna*, we utilized an estimate of the eucalyptus biomass from Ludvichak et al. (2022) as a highly conservative scenario. This approach aligns with the pond model based on maize (Carstens et al., 2012). The maximum leaf concentrations were converted to EECs in the test water based on the model: 0.68 mg/L for Cry1Ab, 0.1 mg/L for Cry1Bb, and 0.12 mg/L for Cry2Aa (detailed calculations in Supplementary Figure S1). For honey bees (detailed calculations in Supplementary Figure S4, S5), the pollen concentrations of 5.08 μg/g for Cry1Ab, 1.24 μg/g for Cry1Bb (LOQ as not detected), and 1.53 μg/g for Cry2Aa were converted to EEDs using pollen intake rates, resulting in 0.0104 μg/g for Cry1Ab, 0.025 μg/g for Cry1Bb, and 0.031 μg/g for Cry2Aa per larva development and 0.0218 μg/g for Cry1Ab, 0.0053 μg/g for Cry1Bb, and 0.0066 μg/g for Cry2Aa per day as per an adult worker (Table 5).

**TABLE 4:** Maximum measured protein expression levels across farms and tree age (µg/g). The **BOLD** highlighted results were used for EED/D estimations.

Tissue	Age	Cry1Ab	Cry1Bb	Cry2Aa
Young leaves	6	51.75	5.8	**9.73**
	12	40.21	4.71	7.27
	24	28.96	3.22	9.38
Mature leaves	6	**53.76**	**8.33**	9.24
	12	30.15	3.07	6.44
	24	18.7	3.26	6.65
Stem	6	21.66	0	1.05
	12	13.72	0	0.51
	24	8.01	0	0.33
Roots	6	7.61	0	0.77
	12	4.59	0	0
	24	2.04	0	0
Pollen	During flowering	**5.08**	0	**1.53**

**TABLE 5:** Calculated values for the no-observed-effect concentration/dose (NOEC/D), estimated environmental concentration/dose (EEC/D), and the margin of exposure (MoE).

Species	Pesticidal protein	NOED µg/larvae	EED µg/larvae	MoE (times)
Honey bee *Apis mellifera* larvae	Cry1Ab	4	0.0104	386
	Cry1Bb	80	0.0025	31,626
	Cry2Aa	136	0.0031	43,573
**Species**	**Pesticidal protein**	**NOED µg/day**	**EED µg/day**	**MoE (times)**
Honey bee *Apis mellifera* adults	Cry1Ab	37	0.0218	1,694
	Cry1Bb	14	0.0053	2,626
	Cry2Aa	18	0.0066	2,736
**Species**	**Pesticidal protein**	**NOEC µg/g of diet**	**EEC µg/g of diet**	**MoE (times)**
Earthworm *Eisenia fetida*	Cry1Ab	2,600	53.76	48
	Cry1Bb	170	8.33	20
	Cry2Aa	2,600	9.73	267
Springtail *Folsomia* *candida*	Cry1Ab	2,600	53.76	48
	Cry1Bb	750	8.33	90
	Cry2Aa	2,600	9.73	267
**Species**	**Pesticidal protein**	**NOEC mg/L**	**EEC mg/L**	**MoE (times)**
*Daphnia magna*	Cry1Ab	30	0.68	44
	Cry1Bb	50	0.1	500
	Cry2Aa	20	0.12	167

The highest concentration or dose of each pesticidal protein that showed no observable effects in the target indicator species was determined. These safe concentrations/doses were used as the NOEC or NOED to calculate the margin of exposure (MoE) for each species (see Table 5). In summary, 30 mg/L for Cry1Ab, 50 mg/L for Cry1Bb, and 20 mg/L for Cry2Aa, in the habitat of *D. magna* for 48 h (Supplementary Table S1), caused no harm and no immobilization in acute tests (OECD, 2004a) similar to the control. When added to the diet of *F. candida*, 2,600 μg/g for Cry1Ab, 750 μg/g for Cry1Bb, and 2,600 μg/g for Cry2Aa (Supplementary Table S2) had similar effects on survival, as did the control in chronic tests (OECD, 2016). Doses of 2,600 μg/g for Cry1Ab, 170 μg/g for Cry1Bb, and 2600 μg/g for Cry2Aa had similar effects on *E. fetida* survival, as did the control (Supplementary Table S3) in chronic tests (OECD, 2004b). Chronic larval toxicity studies with *A. mellifera* (OECD, 2021) showed that exposure to 4 μg Cry1Ab, 80 μg Cry1Bb, or 136 μg Cry2Aa during larval development was safe, with no significant differences in the survival or adult emergence compared to the control (Supplementary Table S4). Ten days of chronic oral exposure of adult bees (OECD, 2017) to 37 μg Cry1Ab, 14 μg Cry1Bb, or 18 μg Cry2Aa per bee per day (Supplementary Table S5) induced no mortality.

The margin of exposure (MoE) ranged from 20 to 43,573 times, indicating that the EEC or EED values were tens to thousands of times lower than the respective NOEC or NOED (Table 5).

### Soil microbial community analysis

**FIGURE 3:**
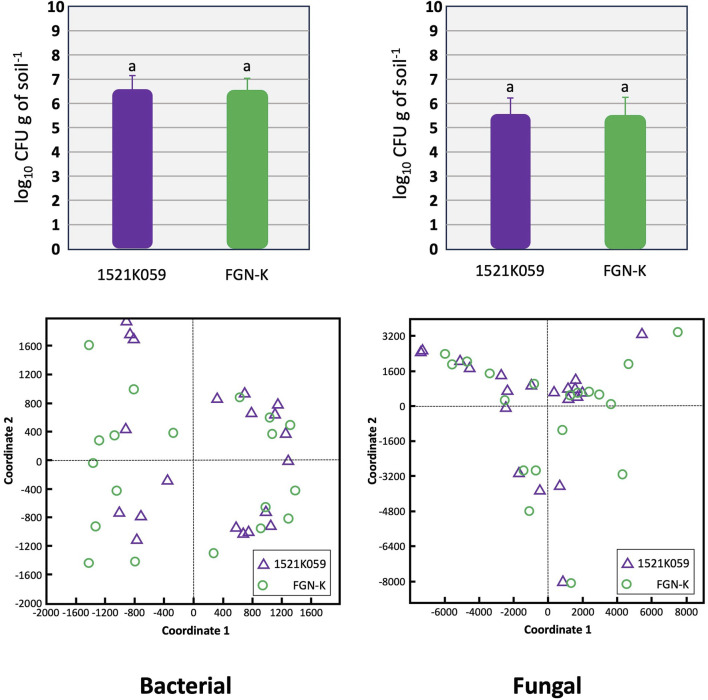
Comparison of the soil microbial composition in plots with the transgenic event 1521K059 versus plots with the wild-type clone FGN-K. Soil samples were collected from plots 24 months after either planting event 1521K059 or the corresponding wild-type clone FGN-K. To evaluate the microbial population diversity based on 16S rRNA sequencing, the principal coordinate analysis (PCoA) was performed using the QIIME alpha diversity pipeline (lower panel). Additionally, colony-forming unit (CFU) analyses were conducted on five biological replicates to assess the microbial densities (upper panel). Both analyses found no significant differences in the microbial diversity or CFUs between 1521K059 and wild-type FGN-K samples (Tukey’s test at the 5% significance level).

Event 1521K059 had no significant impact on the soil microbial community. Microbial assessments conducted 24 months after planting found no notable difference in the composition and the density of bacteria and fungi between plots containing the event 1521K059 vs. those with the wt FGN-K clone (Figure 3 upper panels) (*p* >0.05). The CFU was quite similar across all four tested biomes in Brazil. Furthermore, the PCoA (Figure 3 lower panels) found no correlation between the soil microbial community and the cultivation of the GM event 1521K059 compared to the wt FGN-K clone.

### Arthropod collection and analysis

**FIGURE 4:**
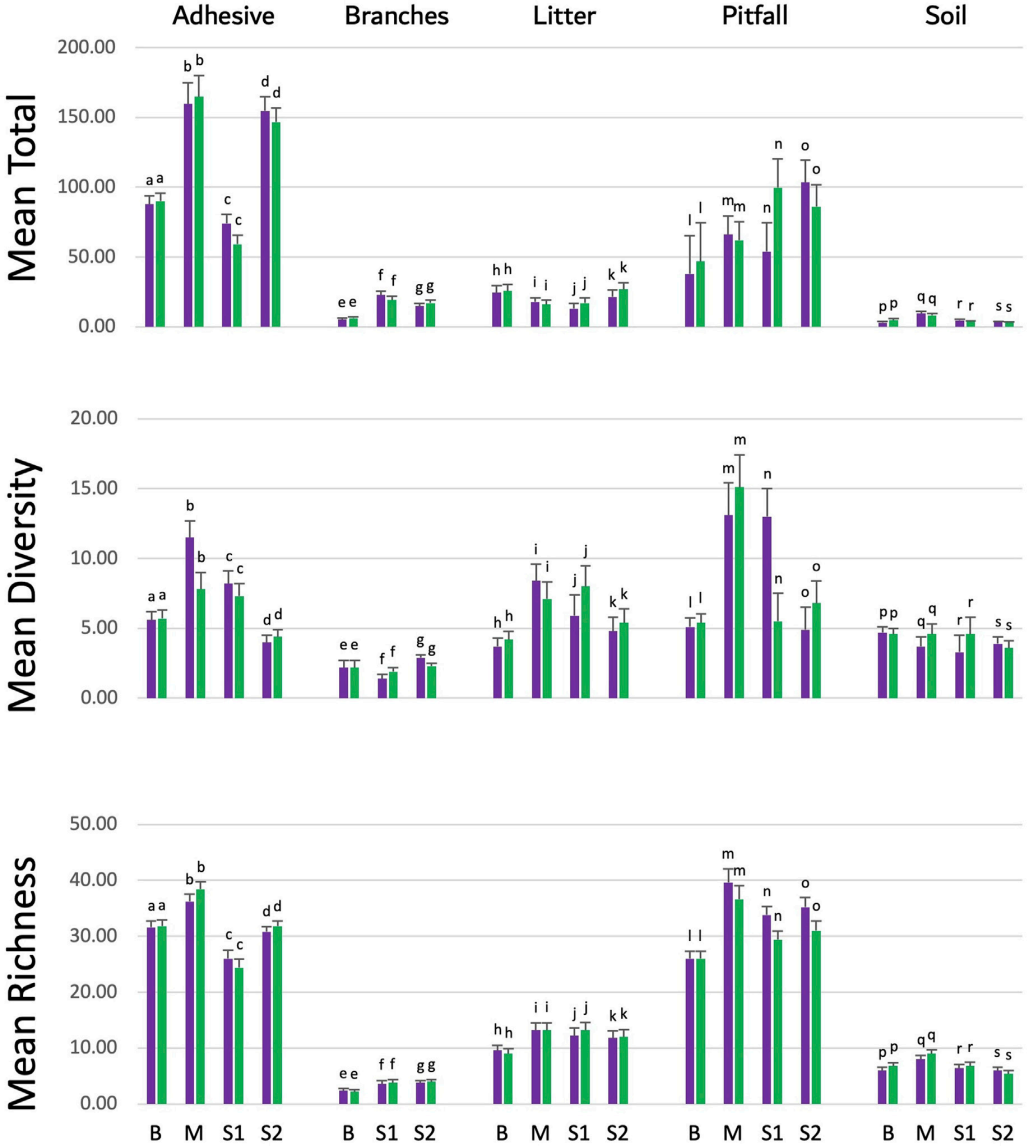
Arthropod richness and abundance in the soil from the transgenic event 1521K059 versus the wild-type clone FGN-K. Arthropod specimens were collected over 2 years from event 1521K059 and wild-type FGN-K plots using pitfall traps, adhesive traps, branch shaking, soil sampling, and litter sampling across four farms (B Bahia, M Maranhão, S1 São Paulo farm 1, and S2 São Paulo farm 2). Samples were analyzed in the laboratory, and the total number of arthropods, richness, and diversity was calculated. Variance analysis with Tukey’s test at the 5% significance level was performed using the agricolae R package (version 1.3.5) in R (version 4.2.1). No significant differences in arthropod richness or abundance were found between 1521K059 and wild-type FGN-K plots across farms and collection methods.

The arthropod populations in areas cultivated with eucalyptus were compared between plots containing the eucalyptus event 1521K059 vs. those with the conventional FGN-K clone, in three different ecosystems across four experimental farms. Branches’ samples from the Maranhão farm were not collected due to technical accessibility issues and were, therefore, not included in the analysis. The results indicated that eucalyptus 1521K059 did not have a significant impact on the arthropod populations inhabiting these areas (Figure 4). For each combination of the collection method (adhesive, branches, litter pitfall, and soil) and parameter (diversity, richness, and the total), the calculated effect size (η^2^) was consistently lower than 0.1, indicating a small effect size (Cohen, 1988; Olejnik and Algina, 2003). These findings were consistent across all five sampling methods (Supplemental Figure S3), indicating that the two eucalyptus varieties had similar effects on the different tested arthropod populations.

### Degradability of the branches and leaves in the field

**FIGURE 5:**
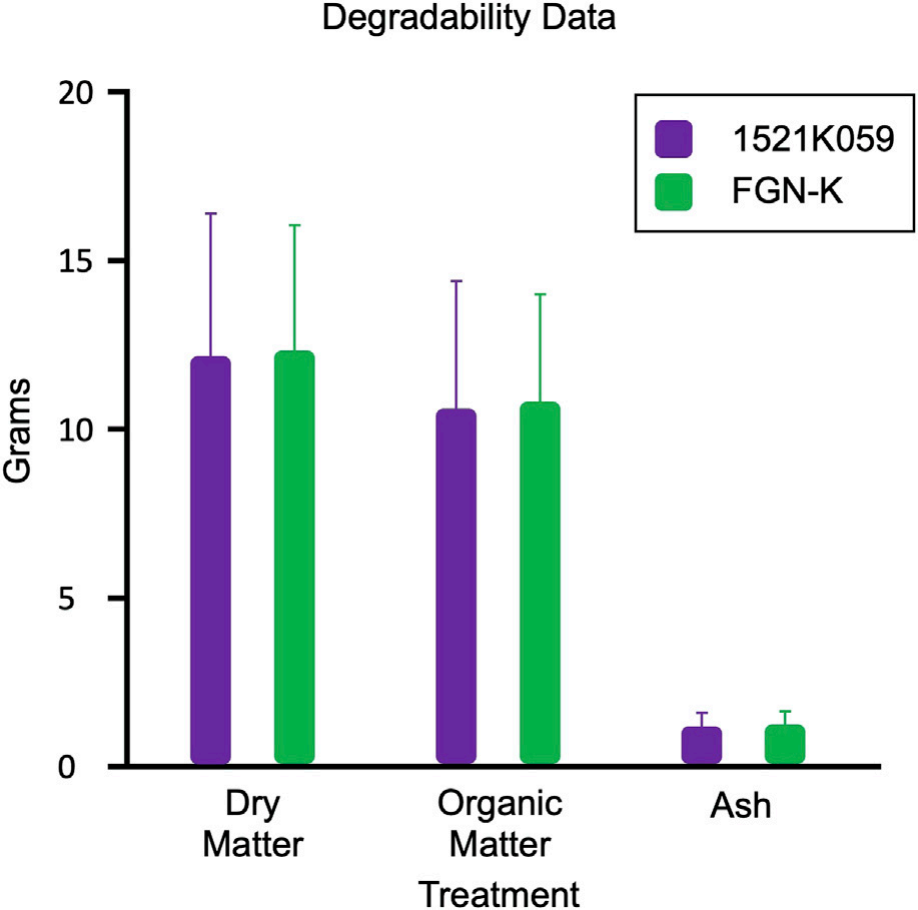
Comparable degradation rates of leaves and branches from the transgenic event 1521K059 and the wild-type FGN-K. The average dry matter, organic matter, and ash weight loss are presented for all four farms. No significant differences were found in the degradability of event 1521K059 compared to the wild-type FGN-K, as determined by Tukey’s test at a 5% significance level.

Over a span of 180 days, the degradability assays found no significant differences in the degradation of the 1521K059 event vs. the FGN-K wt biomass (Figure 5). The relatively high standard deviations can be attributed to the exposure of the bags to field conditions in diverse biomes, where each farm possesses distinct characteristics, such as varying levels of precipitation, soil composition, humidity, and temperatures. These variations can influence the observed degradation rate on each farm (Ribeiro et al., 2018), and the site effect analysis can be seen in Supplemental Figure S7. At all sites, the transgenes and genetic modifications have no impact on the degradability of eucalyptus tissues in the soil.

## Discussion

This study assessed the biosafety of the first commercially approved GM eucalyptus tree with lepidopteran pest resistance, labeled as event 1521K059. This pioneering Bt eucalyptus variant was engineered to express Cry1Ab, Cry1Bb, and Cry2Aa pesticidal proteins to control *T. arnobia*, one of the primary lepidopteran pests affecting eucalyptus. Each pesticidal protein can independently control the caterpillars, but utilizing the triplet stack enhances the product’s durability and complements integrated pest management (IRM) programs. Detailed activity data for the event and information on the modes of action of these pesticidal proteins will be presented in an upcoming manuscript. The platform is a new tool contributing to the portfolio of biotechnological solutions aimed at assisting farmers in managing the growing threat of pest attacks, reducing the need for pesticides, and increasing the crop yield (Brookes and Barfoot, 2018). *Bt*, recognized as a safe biological pest control agent, along with its pesticidal proteins, had no adverse effects on the environment, human health, or animal welfare. Importantly, experimental data underscored the high specificity of these proteins, as evidenced in tests involving non-target organisms, encompassing both vertebrates and invertebrates (Shimada et al., 2006; Yu et al., 2011; Farias et al., 2015; Wang et al., 2018; Meissle et al., 2022).

*Bt* crop cultivation, adopted in 27 countries, has covered over 100 million hectares of agricultural land since 1995 (ISAAA, 2020; Tabashnik et al., 2023), earning safety approvals for food, feed, and environmental use (Mendelsohn et al., 2003; Koch et al., 2015). It represents the safest technology that is currently available to substitute for chemical pesticides, which pose direct negative consequences for farmers, consumers, non-target organisms, and ecosystems (Brookes, 2022; Yang et al., 2023). The defensive response of eucalyptus trees to feeding pests, which leads to the increased production of lignin and extractives, is known to negatively affect the wood quality (Khattab and Khattab, 2005; Zanuncio et al., 2020). The absence of caterpillar-induced damage is likely to directly augment the wood biomass, while the improved wood quality is projected to substantially increase pulp production (Zanuncio et al., 2020). Thus, insect-resistant Bt eucalyptus offers the potential to significantly enhance the yield and reduce the environmental impact of pest control measures.

Comprehensive biosafety assessments spanning 2 years positioned the insect-resistant Bt eucalyptus at a safety standard equivalent to other commercially established Bt crops, confirming its suitability for responsible farming practices. The *in silico* analysis (based on the datasets from 2022) indicated that the Cry1Ab, Cry1Bb, and Cry2Aa pesticidal proteins are not anticipated to present substantial risks to human health. Tests for protein digestibility and thermostability further corroborate to the safety of these proteins. Similar to other Cry pesticidal proteins that have been assessed and Cry1Ab that was tested before (Okunuki et al., 2002; de luis et al., 2009), Cry1Bb and Cry2Aa lost their immunoreactivity and potential activity at approximately 60°C (Wang et al., 2018) and were degraded when exposed to the SGF but resisted digestion in the SIF alone (Farias et al., 2015). Cry proteins are known to resist degradation in the SIF, but research has shown that if they first encounter gastric fluids, the resulting peptides are completely broken down when they arrive at the simulated intestinal environment (US EPA, 2010). This is crucial as it signifies that under the standard physiological conditions, where proteins are efficiently digested in the stomach first, Cry proteins would be reduced to amino acids or small peptides before reaching the intestines.

Arthropods and soil microbes play an important role in maintaining the ecological balance, including in agricultural fields and farms (Paoletti, 2012; Zhang et al., 2023). Due to their high sensitivity to alterations in crop growth and cultivation practices, they can act as “bioindicators” to identify impacts on specific field ecosystems. A three-year study on four farms investigated whether the genetically modified event 1521K059 impacted the arthropod and soil microbial populations in comparison to the non-modified wild-type clone FGN-K. No significant discrepancies were found between the plots of 1521K059 and FGN-K, suggesting that the introduction of the genetically modified event 1521K059 did not induce ecological alterations. This, along with similar degradation rates of the event 1521K059 and the FGN-K wild-type, suggests that the GM IR eucalyptus poses a low risk to the environment.

Given that eucalyptus products from Brazil are not employed for human or animal consumption, the assessment of the potential toxicity of the pesticidal proteins present in the genetically modified eucalyptus event 1521K059 was conducted in non-target organisms (NTOs), as required by the CTNBio, following the guidelines outlined in the OECD for assessing the potential impacts of chemicals on both human health and the environment (OECD, 2004a; OECD, 2004b; OECD, 2016; OECD, 2017; OECD, 2021). The evaluation encompassed honey bee *A. mellifera*, earthworm *E. fetida*, the springtail *F. candida*, and the aquatic invertebrate *D. magna*, which are the four well-established surrogate species, widely employed for safety analyses and as representatives of other NTOs in the environment. Eucalyptus is known to be attractive to bees (Cham et al., 2017), and exposure to the Bt protein primarily occurs through the consumption of pollen and nectar from eucalyptus flowers. Aquatic invertebrates, such as *D. magna*, may come into contact with the Bt protein through the ingestion of the solubilized protein in water or plant tissues from eucalyptus transported into the bodies of water. The 48-h acute toxicity tests for Bt pesticidal proteins are commonly conducted, as indicated by various studies (Federici and Siegel, 2008; Wolt et al., 2008; ILSI, 2011). Tests assessing the impact of these proteins on lepidopteran targets, which typically require 5–7 days to record 100% mortality, indicate a mortality rate of 20%–40% after 48 h (Babu et al., 2002). In contrast, mosquitoes demonstrate larval mortality ranging from 91.0% to 100% within 24 h (Derua et al., 2022). Following a thorough examination, which revealed no adverse effects, even at high doses of Cry1Ab, Cry1Bb, and Cry2Aa within the initial 48 h, there was no request to extend the *D. magna* tests to cover chronic reproduction (7–21 days). Edaphic fauna, comprising arthropods, like earthworms and springtails, can feed on eucalyptus leaves incorporated into the soil and on possible exudates or decomposed parts of plant roots. The Brazilian legislation does not specify the ecotoxicological studies to be conducted, leaving it to the applicant to determine which studies are most relevant to the environment where the GMO will be planted. The list of the selected indicator organisms was deemed sufficient by the CTNBio, and there is no requirement to conduct studies with predatory arthropods.

The MoE values (Table 5) exceeded the EEC and EED by more than a 10-fold margin and adhered to the NTO testing standards outlined by the U.S. EPA (U.S. EPA, 2007; U.S. EPA, 2010), suggesting the absence of the potential harm to any of these organisms. These MoE values are low-conservative estimate values suggesting worst-case scenarios, and the actual safety margins are potentially higher due to the lower pesticidal protein concentrations in the tree tissues and pollen and the extreme values used for the EEC/EED calculations.

The biosafety assessments of event 1521K059, including molecular characterization, toxicity and allergenicity, and the environmental impact, present a safety profile comparable to that of the conventional eucalyptus clone FGN-K. This supports the conclusion that this genetically modified insect resistant eucalyptus variety is safe for use in wood and fiber production and poses negligible risks to human or animal health or the environment.

The sequencing data on the event 1521K059 revealed a single insertion site in the genome, with no direct impact on any endogenous genes. This insertion can serve as a marker for tracking the events in the future planting and breeding activities. Trait introgression occurs at a slow pace in eucalyptus due to its inherent incompatibility with selfing and backcrossing (Hedrick et al., 2016). Consequently, facilitating the integration of the desired genes into eucalyptus breeding populations across different biomes requires more than a single GM event. Additional parental genetic backgrounds, carrying the genes, are essential for each biome’s breeding population as the genetic background of 1521K059 is not universally suitable for all biomes in Brazil. Moreover, relying on a sole event may lead to linkage drag, connecting with an undesirable locus in the genome. Such outcomes might only become apparent in the years to come, owing to the yet unexplored genetic maps of eucalyptus (Bartholomé et al., 2015). Hence, in order to facilitate parallel breeding and promote sustainability in eucalyptus farms, it will be necessary to deregulate multiple GM events, each varying in their genetic background and/or T-DNA insertion sites. At the same time, an integrated resistance management program is being developed to ensure the durability of this caterpillar-resistant eucalyptus.

## Data Availability

The raw data supporting the conclusion of this article will be made available by the authors, without undue reservation. The T-DNA insertion site sequencing data is available at GenBank OR817761.
