# Supplementary material for: Genetically engineered eucalyptus expressing pesticidal proteins from Bacillus thuringiensis for insect resistance: a risk assessment evaluation perspective
Source: Front Bioeng Biotechnol. 2024 Mar 18;12:1322985. doi: 10.3389/fbioe.2024.1322985 (PMC10982518; doi:10.3389/fbioe.2024.1322985)
Supplement: Supplementary file 1 [file DataSheet1.PDF]

frontiers

Supplementary Material

Genetically engineered eucalyptus expressing pesticidal proteins from Bacillus thuringiensis for insect resistance: a risk assessment evaluation perspective

Dror Avisar*, Co-Author, Co-Author

* **Correspondence:** Corresponding Author: dror@futuragene.com

Figure S1

Cry1Ab amino acid sequence:

MDNNPNINECIPYNCLSNPEVEVLGGERIETGYTPIDISLSLTQFLLSEFVPGAGFVLGLVDIIWGIFGPSQWDAFLVQIEQLINQRIEEFARNQAISRLEGLSNLYQIYAESFREWEADPTNPALREEMRIQFNDMNSALTT
AIPLFAVQNYQVPLLSVYVQAANLHLSVLRDVSVFGQRWGFDAATINSRYNDLTRLIGNYTDHAVRWYNTGLERVWGPDSRDWIRYNQFRRELTLTVLDIVSLFPNYDSRTYPIRTVSQLTREIYTNPVLENFDGSFRGS
AQGIEGSIRSPHLMDILNSITIYTDAHRGEYYWSGHQIMASPVGFSGPEFTFPLYGTMGNAAPQQRIVAQLGQGVYRTLSSTLYRRPFNIGINNQQLSVLDGTEFAYGTSSNLPSAVYRKSGTVDSLDEIPPQNNNVPPR
QGFSHRLSHVSMFRSGFSNSSVSIIRAPMFSWIHRSAEFNNIIPSSQITQIPLTKSTNLGSGTSVVKGPGFTGGDILRRTSPGQISTLRVNITAPLSQRYRVRIRYASTTNLQFHTSIDGRPINQGNFSATMSSGSNLQSGSF
RTVGFTTPFNFSNGSSVFTLSAHVFNSGNEVYIDRIEFVPAEVTFEAEYDLERAQK

Cry1Bb amino acid sequence:

MTSNRKNENEIINALSIPTVSNPSTQMNLSPDARIEDSLCVAEVNNIDPFVSASTVQTGINIAGRILGVLGVPFAGQLASFYSFLVGELWPSGRDPWEIFLEHVEQLIRQQVTENTRNTAIARLEGLGRGYRSYQQALETW
LDNRNDARSRSIILERYVALELDITTAIPLFRIRNEEVPLLMVYAQAANLHLLLLRDASLFGSEWGMASSDVNQYYQEQIRYTEEYSNHCVQWYNTGLNNLRGTNAESWLRYNQFRRDLTLGVLDLVALFPSYDTRTYPIN
TSAQLTREIYTDPIGRTNAPSGFASTNWFNNNAPSFSAIEAAIFRPPHLLDFPEQLTIYSASSRWSSTQHMNYWVGHRLNFRPIGGTLNTSTQGLTNNTSINPVTLQFTSRDVYRTESNAGTNILFTTPVNGVPWARFNF
INPQNIYERGATTYSQPYQGVGIQLFDSETELPPETTERPNYESYSHRLSHIGLIIGNTLRAPVYSWTHRSADRTNTIGPNRITQIPLVKALNLHSGVTVVGGPGFTGGDILRRTNTGTFGDIRLNINVPLSQRYRVRIRYAS
TTDLQFFTRINGTTVNIGNFSRTMNRGDNLEYRSFRTAGFSTPFNFLNAQSTFTLGAQSFSNQEVYIDRVEFVPAEVTFEAEYDLERAQK

Cry2Aa amino acid sequence:

MNNVLNSGRTTICDAYNVVAHDPFSFEHKSLDTIQKEWMEWKRTDHSLYVAPVVGTVSSFLLKKVGSLIGKRILSELWGIIFPSGSTNLMQDILRETEQFLNQRLNTDTLARVNAELIGLQANIREFNQQVDNFLNPTQ
NPVPLSITSSVNTMQQLFLNRLPQFQIQGYQLLLLPLFAQAANMHLSFIRDVILNADEWGISAATLRTYRDYLRNYTRDYSNYCINTYQTAFRGLNTRLHDMLEFRTYMFLNVFEYVSIWSLFKYQSLMVSSGANLYASG
SGPQQTQSFTAQNWPFLYSLFQVNSNYILSGISGTRLSITFPNIGGLPGSTTTHSLNSARVNYSGGVSSGLIGATNLNHNFNCSTVLPPLSTPFVRSWLDSGTDREGVATSTNWQTESFQTTLSLRCGAFSARGNSNYFP
DYFIRNISGVPLVIRNEDLTRPLHYNQIRNIESPSGTPGGARAYLVSVHNRKNNIYAANENGTMIHLAPEDYTGFTISPIHATQVNNQTRTFISEKFGNQGDSLRFEQSNTTARYTLRGNGNSYNLYLRVSSIGNSTIRVTIN
GRVYTVSNVNTTTNNDGVNDNGARFSDINIGNIVASDNTNVTLDINVTLNSGTPFDLMNIMFVPTNLPPLY

Figure S1 – Amino acid sequences. Of Cry1Ab, Cry1Bb and Cry2Aa, that were used and are translated from the DNA of event 1521K059.

Figure S2

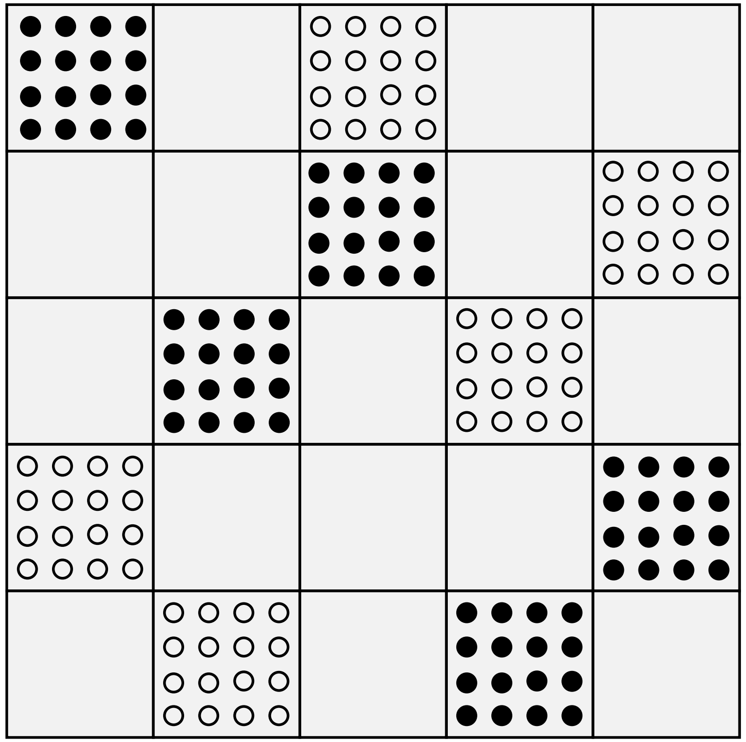

Figure S2, Field trials design. Eucalyptus event 1521K059 (black dots) and the wild-type (wt) control clone FGN-K (white dots) were planted at four sites in Brazil: two in the State of São Paulo (SP), one in the State of Bahia (BA), and one in the State of Maranhão (MA). The planting design consisted of square plots, each containing 16 plants. Five square plots of each clone/event were randomly distributed in blocks within the field, alongside other plots of unrelated clones that were not part of the experiments.

Figure S3

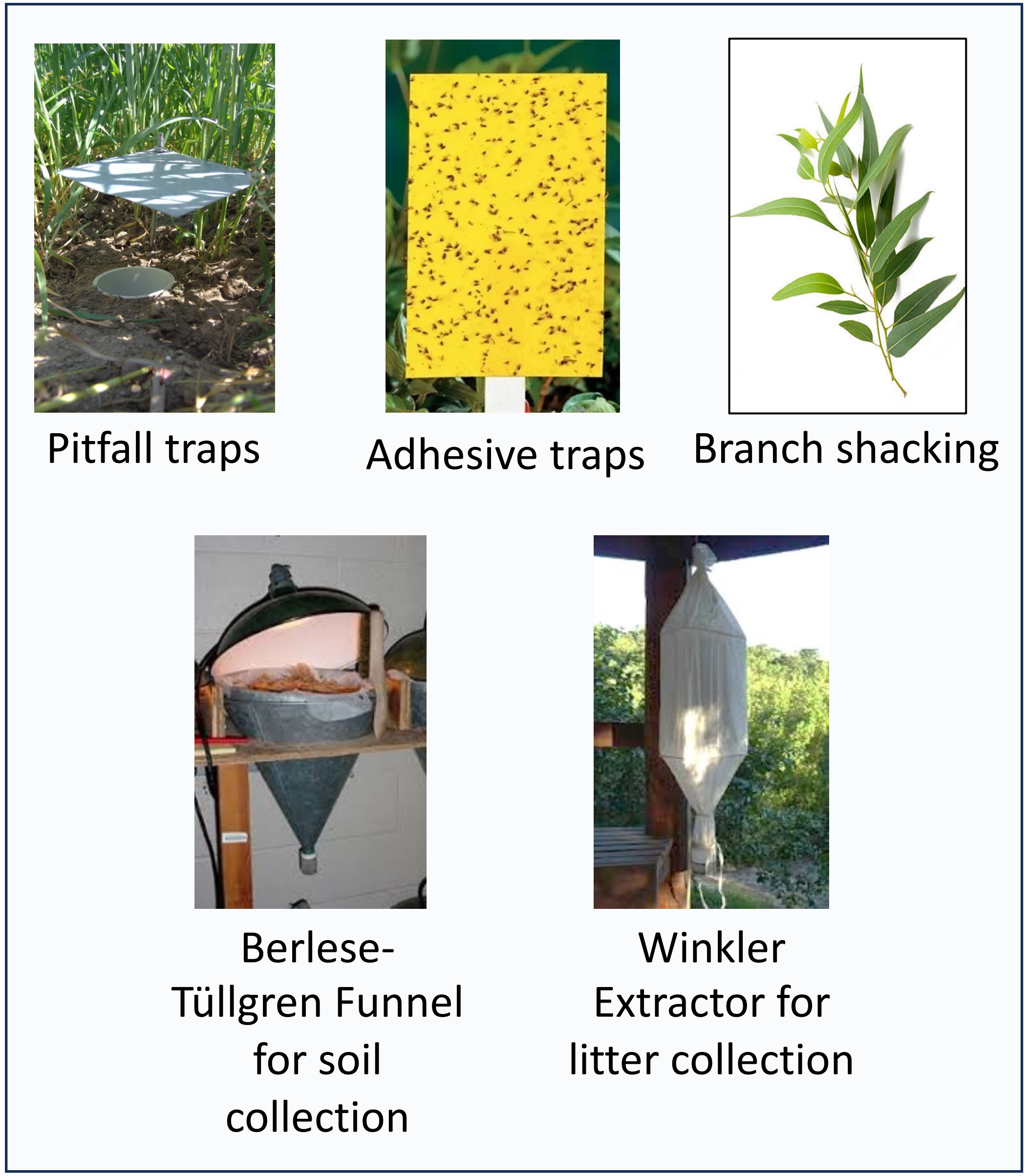

Figure S3: The five arthropod collection methods

Figure S4

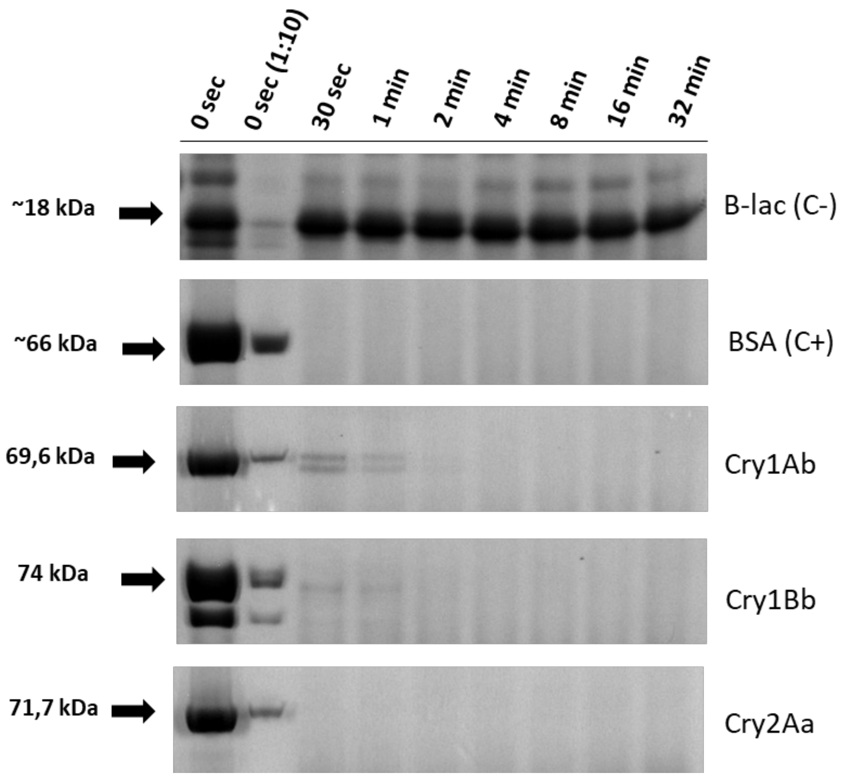

Figure S4: SDS-PAGE gel showing the analysis of proteins β-lac (M.W. ~18 kDa), BSA (M.W. ~66 kDa), Cry1Ab (M.W. 69.6 kDa), Cry1Bb (M.W. 74 kDa), and Cry2Aa (M.W. 71.7 kDa) subjected to digestion by Simulated Gastric Fluid (SGF). β-lac resist digestion in SGF while BSA and the other Cry pesticidal protein showed high digestibility in SGF starting at 30 sec.

Figure S5

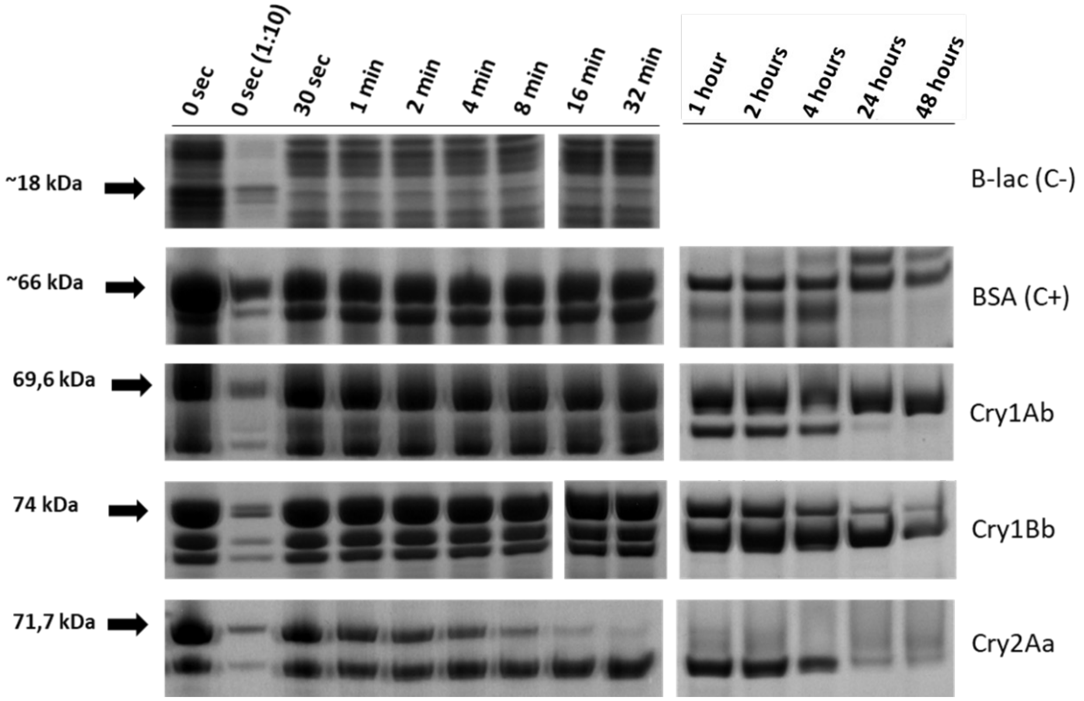

Figure S5: SDS-PAGE gel analysis of proteins β-lac (M.W. ~18 kDa), BSA (M.W. ~66 kDa), Cry1Ab (M.W. 69.6 kDa), Cry1Bb (M.W. 74 kDa), and Cry2Aa (M.W. 71.7 kDa) subjected to digestion by Simulated Intestinal Fluid (SIF). BSA showed high digestibility in SGF while β-lac and the other Cry pesticidal protein resist digestion in SIF for 2-48 hours.

Figure S6

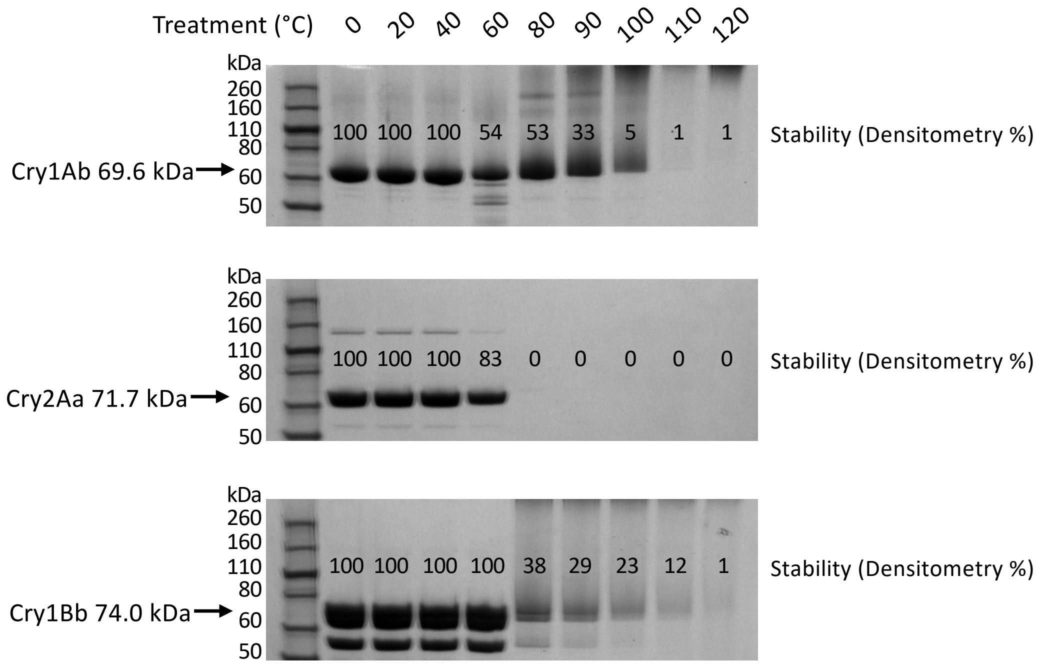

Figure S6: The thermal stability of Cry1Ab, Cry1Bb, and Cry2Aa pesticidal proteins was assessed by subjecting them to increasing temperatures (20, 40, 60, 80, 90, 100, 110, and 120 °C) for 20 min. The soluble portions were analyzed by SDS-PAGE. The results showed that Cry1Ab (upper panel), Cry2Aa (middle panel) and Cry1Bb pesticidal protein (lower panel) were degraded as the temperature increased, as indicated in Table 3. Densitometry used BioRad's Image Lab software version 6.1 to compare the size and intensity of the protein bands with those observed following 0 °C treatment (control).

Figure S7

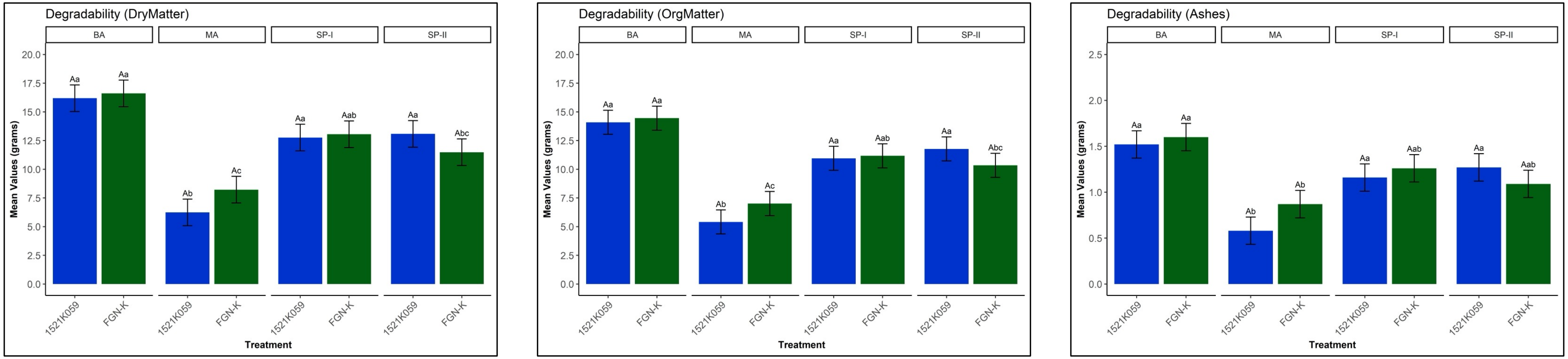

Figure S7: Degradability of branches and leaves in the field by farm sites – expansion of figure 5. No significant differences were found in the degradability of event 1521K059 compared to wild-type FGN-K. Uppercase letters represent statistical significance within the farm and lowercase letters represent statistical significance between the farms (Tukey multiple comparison test with a significance level of 5%).

Figure S8

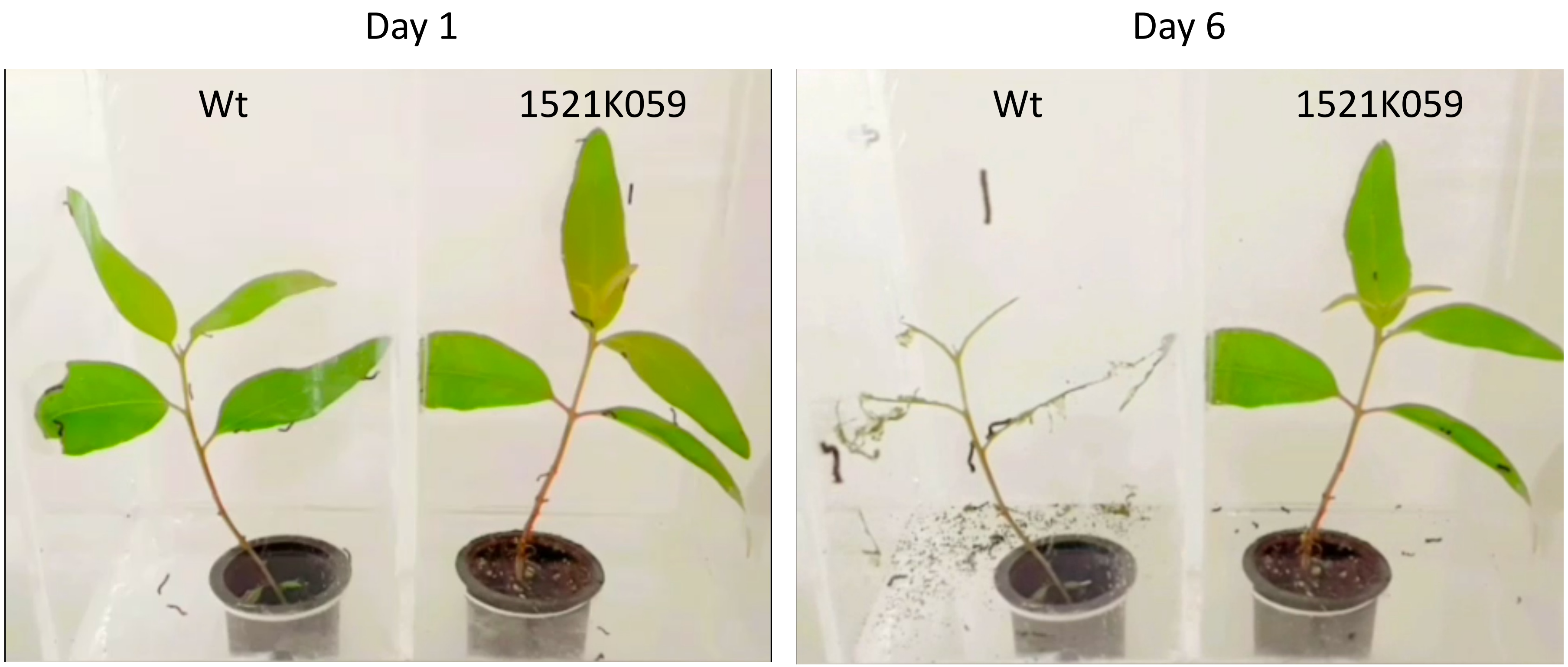

Figure S8: The genetically modified (GM) eucalyptus plant (event 1521K059) on the left and a wild type eucalyptus plant (FGN-K) on the right. Ten second instar *T.arnobia* caterpillars were placed in a cage with a 3-month-old plantlet of each type. After 6 days, the GM eucalyptus event 1521K059 had no damage, while the leaves of the WT eucalyptus plant were completely eaten by the larvae.

Table S1

48-Hour Exposure of Daphnids (*Daphnia magna*) to pesticidal proteins - Nominal Concentrations Tested, Corresponding Cumulative Percent and Number of Immobilized Organisms										
**Pesticidal protein nominal concentration**	**Percent Immobilization**									
	**24 Hours**					**48 Hours**				
	**A**	**B**	**C**	**D**	**Mean**	**A**	**B**	**C**	**D**	**Mean**
**Control**	0	0	0	0	0	0	0	0	0	0
**Cry1Ab 30 mg/L**	0	0	0	0	0	0	0	0	0	0
**Cry1Bb 50 mg/L**	0	0	0	0	0	0	0	0	0	0
**Cry2Aa 20 mg/L**	0	0	0	0	0	0	0	0	0	0
These values represent the average cumulative mortality of four replicates (A, B, C and D) in each treatment group. 5 Daphnids per replicate.										

EEC calculation:

25,200 kg/hectare X 10 hectare = 252,000 Kg of Eucalyptus tisues
252,000 Kg / 20,000,000 Litters = 0.0126 Kg of eucalyptus tissues/Litter

Cry1Ab concentration is 53.76 µg/g = 53.76 mg/kg
Cry1Ab EEC = 0.0126 kg/L of eucalyptus tissue X 53.76 mg/Kg = 0.68 mg/L

Cry2Aa concentration is 9.73 µg/g = 9.73 mg/kg
Cry2Aa EEC = 0.0126 kg/L of eucalyptus tissue X 9.73 mg/Kg = 0.12 mg/L

Cry1Bb concentration is 9.73 µg/g = 8.33 mg/kg
Cry1Bb EEC = 0.0126 kg/L of eucalyptus tissue X 8.33 mg/Kg = 0.1 mg/L

Table S2

**Chronic Exposure of Springtails (*Folsomia candida*) to pesticidal proteins - Mean Percent Survival**					
**Pesticidal protein concentration**	**Percent Survival (Day 28)**				
	**A**	**B**	**C**	**D**	**Mean (SD)**
**Control**	90	90	100	70	88 (13)
**Cry1Ab 2600 µg/g**	100	80	90	80	88 (10)
**Cry1Bb 750 µg/g**	100	80	80	80	85 (10)
**Cry2Aa 2600 µg/g**	70	100	100	90	90 (14)

No statistically significant differences in survival were identified compared to the controls, based on Fisher's Exact Test with Bonferroni-Holm adjustment. These values represent three replicates (A, B, C and D) with 10 Juvenile springtails per replicate.

Table S3

**Chronic Exposure of Earthworms (*Eisenia fetida*) to pesticidal proteins - Mean Percent Survival**					
**Pesticidal protein concentration**	**Percent Survival (Day 28)**				
	**A**	**B**	**C**	**D**	**Mean (SD)**
**Control**	90	100	100	100	98 (5)
**Cry1Ab 2600 µg/g**	100	100	100	90	98 (5)
**Cry1Bb 170 µg/g**	100	100	100	100	100 (0)
**Cry2Aa 2600 µg/g**	90	100	100	100	98 (5)

No statistically significant differences in survival were identified compared to the controls, based on Fisher's Exact Test with Bonferroni-Holm adjustment. These values represent three replicates (A, B, C and D) with 10 Adult earthworms per replicate.

Table S4

**Chronic Larval Toxicity Study with the Honey Bee (*Apis mellifera*)**				
**Pesticidal protein per larval development**	**Percent Survival and Adult Emergence**			
	**A**	**B**	**C**	**Mean (SD)**
**Control**	79	93	64	79 (15)
**Cry1Ab 4 µg**	71	93	79	81 (11)
**Control**	93	93	79	88 (8)
**Cry1Bb 80 µg**	64	86	93	81 (15)
**Control**	100	100	71	90 (17)
**Cry2Aa 136 µg**	93	100	93	95 (4)

No statistically significant differences in survival were identified compared to the controls, based on Shapiro-Wilk's and Lavente's tests (α=0.05). These values represent three replicates (A, B and C) with 14 Larvae per replicate.

EED calculation:

Cry1Ab in pollen = 5.08 µg/g = 0.00508 µg/mg
Cry1Ab EED = 2.04 mg pollen/Larvae X 0.00508 µg/mg = 0.0104 µg/Larvae

Cry1Bb in pollen = 1.24 µg/g = 0.00124 µg/mg
Cry1Bb EED = 2.04 mg pollen/Larvae X 0.00124 µg/mg = 0.0025 µg/Larvae

Cry2Aa in pollen = 1.53 µg/g = 0.00153 µg/mg
Cry2Aa EED = 2.04 mg pollen/Larvae X 0.00153 µg/mg = 0.0031 µg/Larvae

Table S5

Daily Mortality of Adult Honey Bees after Chronic Oral Exposure to Pesticidal Protein										
**Pesticidal protein**	**Average Mortality (%) on Day:**									
	**1**	**2**	**3**	**4**	**5**	**6**	**7**	**8**	**9**	**10**
**Control**	0	0	0	0	0	0	0	0	0	0
**Cry1Ab 37 µg/day**	0	0	0	0	0	0	0	0	0	0
**Cry1Bb 14 µg/day**	0	0	0	0	0	0	0	0	0	0
**Cry2Aa 18 µg/day**	0	0	0	0	0	0	0	0	0	0
These values represent the average cumulative mortality of three replicates in each treatment group. 10 Bees per replicate.										

EED calculation:

Cry1Ab in pollen = 5.08 µg/g = 0.00508 µg/mg
Cry1Ab EED = 4.3 mg pollen/day X 0.00508 µg/mg = 0.0218 µg/day

Cry1Bb in pollen = 1.24 µg/g = 0.00124 µg/mg
Cry1Bb EED = 4.3 mg pollen/day X 0.00124 µg/mg = 0.0053 µg/day

Cry2Aa in pollen = 1.53 µg/g = 0.00153 µg/mg
Cry2Aa EED = 4.3 mg pollen/day X 0.00153 µg/mg = 0.0066 µg/day
